# Applying the 5Cs Framework to Elite Youth Tennis: Impact Factors in a Talent Development Environment

**DOI:** 10.3390/bs16020166

**Published:** 2026-01-25

**Authors:** Chris Harwood, Kieran Porter

**Affiliations:** 1 Department of Sport Science, Nottingham Trent University, Nottingham NG1 4FQ, UK; 2 Rounded Performance Consultancy Ltd., Leicestershire LE9 9JJ, UK; kieran@roundedperformance.com

**Keywords:** parents, athletes, coaches, youth tennis, psychosocial development

## Abstract

With the growing demands and expectations associated with professionalised youth sport environments, there is an increasing need for psychosocial development initiatives to support young athletes and their healthy progression. The aim of this study was to extend and investigate the application of the 5Cs framework, a prominent psychoeducational approach in sport psychology, to a youth tennis Talent Development Environment (TDE). Using a collective case study design, five athletes, their parents and two coaches (*n* = 12) participated in a season-long multimodal 5Cs intervention programme at a British Regional Player Development Centre (RPDC). The 30-week programme was delivered by an embedded sport psychology practitioner (SPP) and incorporated a blocked educational curriculum supported by a range of athletes, coach and parent development strategies. Post-intervention semi-structured interviews were conducted with all participants, with reflexive thematic analysis leading to three overarching themes. Findings highlighted the positive influence of the programme, with perceptions of the framework’s effectiveness associated with its specificity to tennis and individual athlete needs, the collaboration of all stakeholders across the TDE and the use of developmentally accessible and innovative strategies enabling evidence of athlete improvements. Researchers, practitioners and sport organisations are encouraged to consider these impact factors in terms of supporting the development, performance and well-being of athletes and their families in competitive youth sport contexts.

## 1. Introduction

Youth sport environments offer challenging and valuable learning experiences for junior athletes, whereby such experiences can promote psychological, physical, emotional, social and intellectual growth (Dorsch et al., 2022; Fraser-Thomas et al., 2005; Thrower et al., 2024). However, each young athlete’s ability to develop and progress within their sporting environments depends on their use of psychosocial strategies and an awareness of characteristics necessary to cope with the demands of their sport (Dohme et al., 2019; Henriksen et al., 2014). This is particularly salient for athletes on elite developmental pathways where, increasingly, professionalised youth sport environments (i.e., training, competition; Harwood & Thrower, 2019) impose significant demands and expectations. The development and application of these psychological strategies and characteristics do not materialise through youth sport participation alone, instead requiring intentional, behaviourally focused and developmentally appropriate training activities supported by key stakeholders, including sport psychology practitioners (SPPs), parents, coaches and peers (see Arthur et al., 2019; Dohme et al., 2019; Sheridan et al., 2014; Steptoe et al., 2019; Thrower et al., 2024).

The body of science related to psychological applications and interventions with young athletes, or within youth sport environments, reflects both limitations and opportunities. Harwood and Thrower (2019) reviewed the literature on performance-focused psychological interventions in youth sports, noting the limited field-based and sport-specific research around which strategies (e.g., goal setting, relaxation, imagery) were most efficacious at different stages of athlete development. They also affirmed that the vast majority of intervention studies in youth sport had been delivered by applied researchers or practitioners, neglecting the equally direct role that coaches (and other social agents) could play with respect to facilitating intervention effectiveness. Further, they noted the emergence of more holistic approaches to psychological and life skills development in young people that could serve to sustain wider psychological health and quality of sport experience, as much as positively impact athletic performance (Dohme et al., 2019; Holt et al., 2017; MacNamara et al., 2010a). Approaches such as the Psychological Characteristics for Developing Excellence model (PCDEs; MacNamara et al., 2010a, 2010b) and the systematic review of studies targeting psychosocial characteristics, conducted by Dohme et al. (2019), illustrated a plethora of characteristics, attributes and strategies believed to be necessary for personal development, athletic progression and performance excellence. Representing a mix of attributes and strategies, including elements such as resilience, coping with pressure, quality practice, competitiveness, imagery, planning and organisation, this work offered sport psychology practitioners (SPPs) a wider psychoeducational blueprint of the concepts they might integrate into their work within talent development environments.

One particular psychoeducational approach that has proved influential in youth sport has been the 5Cs Framework developed by Harwood (2008) through his consulting work in professional youth football academies. The framework targets the development and demonstration of five psychosocial competencies through attention to desired athlete behaviours aligned to the sport. The 5Cs (Commitment, Communication, Concentration, Control and Confidence; see Harwood, 2008; Harwood & Anderson, 2015) capture the motivational, interpersonal, attentional and self-regulatory concepts rooted in established theories of talent development. For example, a focus on Commitment draws upon the salience of fostering a young athlete’s task-involvement and intrinsically motivated behaviours aligned to the principles of Achievement Goal Theory (Nicholls, 1989) and Self-Determination Theory (Deci & Ryan, 1985).

As an embedded scientist–practitioner in a UK youth football academy, Harwood (2008) intervened directly with coaches in order for them to systematically deliver training sessions intentionally focused on promoting and reinforcing 5C behaviours in players. His 5C coach development programme incorporated cycles of coach education, ‘on pitch’ field-based application and evaluation. Coaches were sequentially introduced to each ‘C’ in separate 90 min workshops where discussion was engaged around core principles and sport-relevant behaviours associated with that ‘C’. Coaches then debated the coaching behaviour, drills/exercises and session management techniques that would influence players’ psychosocial behaviour within a training session. Coaches were also introduced to eight pedagogical directives, forming the mnemonic ‘PROGRESS’ (Harwood & Anderson, 2015), that provided a compass for coaches to intentionally promote the ‘C’ to players and engage specific drills, reinforcement and peer support strategies that would stimulate target behaviours.

Harwood’s (2008) field work improved psychosocial coaching efficacy—the confidence coaches possessed in their coaching practice towards influencing a player’s psychosocial competencies. This was supported by the coach’s perceived improvements in players’ psychosocial (i.e., 5Cs) responses and behaviour in training sessions over the course of the three-month intervention. In a follow-up multiple baseline, single-case design study, Harwood et al. (2015) advanced the methodology by collecting data on football players’ self-perceptions, coach perceptions and parents’ perceptions of their sons’ 5C behaviours in training situations. A targeted U-13s age group coach received sequential education in the 5Cs framework prior to integrating relevant strategies across the five intervention phases. Results indicated cumulative increases in positive psychosocial responses across the intervention for selected players, with these improvements corroborated by parent and coach data alongside post-intervention interviews.

Due to the value of promoting a shared and common language towards psychosocial development across environmental stakeholders, the 5Cs framework has since been extended beyond coach-to-player interactions in football. Interventions have incorporated coach observations, player-centredpsychoeducation and player-led team talks (Mitchell et al., 2022, 2024), while academy parents exposed to a 5Cs education curriculum have noted positive adaptations to their parent–athlete interactions (Kramers et al., 2023). In sum, research and practice within football Talent Development Environments (TDEs) have demonstrated the utility of the 5Cs framework as a unifying and collaborative approach to psychosocial development that benefits from the alignment of multiple stakeholders (see Steptoe et al., 2019). However, there remains a paucity of research into the framework’s applicability outside of academy football, and no investigation of its application in individual sports.

Individual youth sport athletes who could benefit heavily from structured attention to their psychosocial competencies are youth tennis players. Youth tennis represents a context rich in serving out physical, psychological, social and emotional challenges on a point-by-point basis. Players compete alone in a punishing scoring system, with restrictions on feedback and requirements to self-officiate, while under pressure to gain results and ranking points for pathway progression (Horne et al., 2020; Lewis et al., 2017). A feature of the youth tennis environment believed to be essential to co-navigating the demands of the sport is the quality of relationships between parents, athletes [i.e., players] and coaches (PACs). Indeed, research within youth tennis illustrates that PACs work more effectively where members communicate openly, establish and pursue shared goals within collaborative and adjustable roles and enact clearly defined responsibilities (Horne et al., 2020; Tagliavini et al., 2024; Wolfenden & Holt, 2005). When communication breaks down, tennis parents can experience stress and uncertainty concerning their child’s development needs (Harwood & Knight, 2009; Horne et al., 2020). Similarly, coaches experience stress to appease parents, particularly when managing their poor tennis understanding and developmentally unrealistic expectations (Knight & Harwood, 2009).

Youth tennis environments represent a valuable opportunity for getting PACs ‘on the same page’ with respect to nurturing important psychosocial qualities from an early age. Yet there are few published accounts of programmes integrating parents, athletes and coaches in youth tennis which systematically apply an established framework to enable psychosocial development (see Dohme et al., 2020; Lauer et al., 2020 for partial examples).

### The Present Study

Evidence for the value of implementing psychological interventions in youth sport is clear (Harwood & Thrower, 2019), but our understanding of how to most effectively deliver such interventions in talent development programs with multiple stakeholders remains restricted. Supporting this challenge, Thrower et al. (2023) gained the insights of highly experienced practitioners around how they sought to optimise development, well-being and performance in their day-to-day work with young athletes. These practitioners used flexible and adaptable approaches, spent time on relationship-building with athletes, coaches and parents in the system and delivered indirect interventions to athletes (through coaches and parents). Such interventions were viewed as most impactful when concepts and strategies were integrated into training and creatively adapted. These insights into the ‘craft of sport psychology’ in youth sport were echoed further by a recent British Psychological Society position on the topic (see Thrower et al., 2024).

With the literature in youth sport at this current juncture, the purpose of the present study was to extend the application and integration of the 5Cs framework into a high-performance youth tennis TDE. Through a season-long multimodal intervention program, this collective case study appraises the utility of the 5Cs Framework as a means of supporting desirable behaviour change in young players through the co-operating influences of their coaches and parents. By investigating participant experiences, the study seeks to understand the critical impact factors underpinning the success of a programmefocused on developing players’ psychosocial characteristics. As such, the research question pursued here is the following: What critical impact factors drive and define an (in)effective 5Cs psychosocial development programme implemented within a high-performance youth tennis environment?

## 2. Method

### 2.1. Philosophical Position and Research Design

The current study was conducted from a pragmatic philosophical perspective. Pragmatists use research findings to solve practical “real-world” problems and improve the human experience. In this respect, pragmatists adopt a relativist ontological position (i.e., truth is what works at the time) and focus on the practical consequences and outcomes of inquiry (Creswell & Creswell, 2019; Poucher et al., 2019). Noting how knowledge is shaped through active engagement and refined through practical outcomes, we were interested in how participants’ engagement with 5Cs-related definitions, use of 5Cs language and psychoeducational strategies in the tennis TDE contributed to their beliefs about the utility of the framework and its role in psychosocial development. As evident within participants’ accounts of their experiences (Raskin, 2008), we also sought to explore the shared perceptions of parents, coaches and athletes towards effective and ineffective programme features through their interactions in the TDE.

In line with this approach, a collective case study design was used as a guiding methodology. A case study offers “an in-depth exploration from multiple perspectives of the complexity and uniqueness of a particular project, policy, institution, programme or system in a ‘real-life’ context” (Simons, 2009, p. 21). Although case study designs do not provide a prescriptive guide for how to collect, analyse and interpret data, the key principles include: (a) small N, (b) contextual detail, (c) everyday setting, (d) boundness (i.e., a detailed description of a temporal or structural boundary, which brings context to the phenomenon being studied), (e) working research question, (f) multiple data sources, and (g) extendibility (see VanWynsbergh & Kahn, 2007). In this study, our participant cases were the coaches, parents and their children contextually bound together by time and space in a single high-performance tennis academy environment. This allowed the researchers to gather in-depth, detailed accounts from the family units and coaches about the 5Cs intervention programme delivered in that specific TDE.

### 2.2. The Research Team

The first author is a White male Health and Care Professions Council (HCPC)-registered sport and exercise psychologist in the UK. As a scientist–practitioner, experienced in qualitative and mixed methods research, he has researched and consulted within the field of youth sport for 32 years. With knowledge of elite tennis settings and the 5Cs framework, he led the study design, write-up and manuscript completion, while contributing to the intervention design and analysis. The second author is a White male SPP who was completing doctoral-level research as a postgraduate prior to successful registration as a HCPC practitioner and sport and exercise psychologist (British Psychological Society, 2024). As part of his independent consultancy practice, he provided embedded support as the SPP within the tennis TDE on two half-days per week to coaches, athletes and parents. He led the intervention delivery, data collection and analysis and contributed to the write-up and manuscript completion.

### 2.3. Participants and Research Context

The collective case study comprised twelve participants (five players, five parents and two coaches) engaged in one British Lawn Tennis Association (LTA) Regional Player Development Centre (RPDC). RPDCs are dedicated to developing ‘nationally relevant’ talent by coaching and supporting gifted junior players aged 10–14 years (Lawn Tennis Association, 2023). Participating athletes accessed the centre on a part-time to full-time basis, training between 7 and 23 h per week. To enable a rich data set, the second author used their familiarity with the programme population to purposefully recruit participants across age groups, gender and in relation to their differing degrees of engagement and time-commitment to the programme (Palinkas et al., 2015). All members of selected parent–athlete–coach (PAC) triads were recruited, including the two participating coaches who had 20 years of coaching experience combined. Both coaches worked with all participating athletes and held LTA Level 5 Master Performance Coach Awards, the highest level coaching qualification in the UK. To ensure anonymity, we have used pseudonyms throughout alongside the demographic case data for family units (i.e., athlete/parent) and coaches (see Table 1).

### 2.4. The 5Cs Psychosocial Development Program

**Figure 1:**
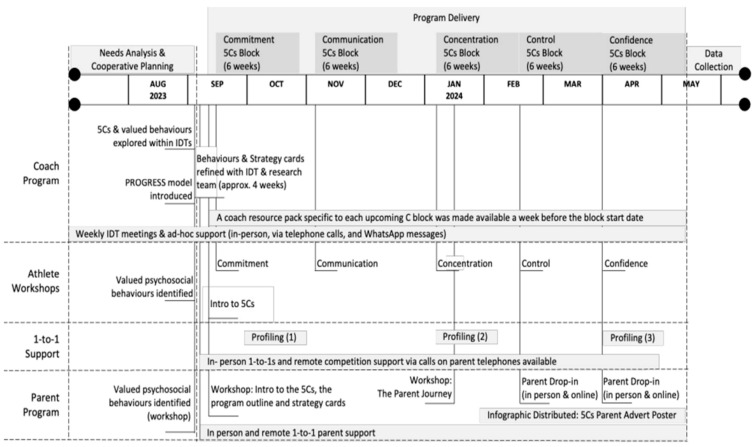
Schematic of the 5Cs programme delivery.

The design and implementation of the 5Cs programme was based on the psychoeducational procedures shared in football academy environments (Harwood, 2008; Harwood & Anderson, 2015; Harwood et al., 2015; Kramers et al., 2023; Steptoe et al., 2019), tailored to the known demands of tennis (Dohme et al., 2020; Harwood & Knight, 2009; Horne et al., 2020; Lauer et al., 2020; Lewis et al., 2017), and the developmental stage of the athletes (Thrower et al., 2024). The multimodal development programme comprised a planned thirty-week, squad-level curriculum, a coach education program, a parent development program, on-court training support and individualised athlete support. Delivered during term times between September 2023 and May 2024, the following subsections detail the key elements and strategies that formed the program, including behavioural techniques and co-design features (see Figure 1 for a full schematic of the 5Cs program).

### 2.5. Programme Planning

**Figure 2:**
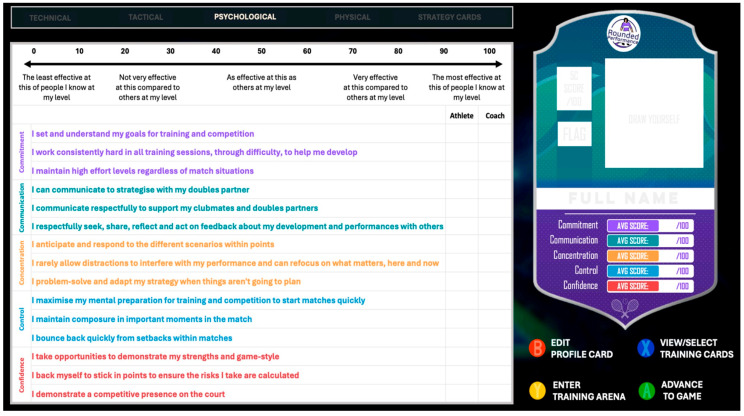
5Cs profiling form to facilitate athlete self and coach assessments.

Commencing the first month of the program, the RPDC coaches, athletes and parents were involved in cooperatively informing the structure and content of the squad-level initiative with the SPP. Through a series of Interdisciplinary Team (IDT) meetings, a player workshop and a parent workshop, the SPP garnered perspectives on the specific psychosocial behaviours and characteristics deemed valuable to athletes in tennis and the support needs of coaches and parents. As a critical first step, the SPP then worked with the coaches and wider research team to refine a final list of agreed psychosocial behaviours aligned and categorised appropriately to each ‘C’ concept. This ultimately informed the Centre-wide conceptualisation of each ‘C’ and an assessment [i.e., 5Cs profiling] tool that would underpin athlete and coach perceptions of athlete psychosocial strengths and areas for development (see Figure 2 for the list of ‘C’-related behaviours; Steptoe et al., 2019).

### 2.6. The Squad-Level Curriculum

The 5Cs programme was integrated into twice-weekly on-court RPDC-squad training sessions with coaches and the SPP. On-court delivery spanned five six-week curriculum blocks, each of which ‘PROGRESS’ed one ‘C’ at a time (Harwood & Anderson, 2015), where coaches integrated on-court education, modelling and reinforcement strategies related to enhancing players’ psychosocial behaviour. In an initial player workshop, the SPP introduced the 5Cs framework, including an exercise that challenged players to build a ‘Frankenstein’s Monster’ of 5C professional tennis role models who demonstrated positive psychosocial qualities. Then, beginning with block 1 on Commitment, the SPP commenced the week with a 30 min psychoeducational workshop. This workshop clarified the RPDC’s shared definition and behaviours associated with that ‘C’ (see Figure 2). It highlighted professional sport role models within and outside tennis, and then explored practical strategies and an experiential learning task to strengthen how players could ‘grow the C’. For example, players completed a “Price and Prize” exercise to understand Commitment, which clarified their tennis goals (the prize) alongside what they were willing to commit to achieve this (the price). At the end of each specific ‘C’ block, parents, athletes and coaches (PACs) were encouraged to continue monitoring and reinforcing the ‘C’ behaviours, even though the on-court focus moved to a new ‘C’. The coaches attended these workshops to provide tennis-related content, inform the alignment of on-court sessions and increase their confidence in reinforcing strategies.

### 2.7. The Coach Education Program

Coach education was initiated through two IDT meetings, which introduced Harwood and Anderson’s (2015) PROGRESS model to staff. Coaches assessed which PROGRESS strategies they delivered effectively and requested support to strengthen others. This self-reflection process informed the psychoeducational content comprising subsequent coach resource packs distributed by the SPP before each ‘C’ block. Each pack defined the ‘C’, explored its underpinning theory (e.g., achievement goal theory) and listed valued behaviours to nurture in players. It then offered guidance on using the PROGRESS model to shape and reinforce the ‘C’ within on-court sessions, including a lexicon of words and phrases that coaches could apply in training. The SPP also shared individual athlete strategies (e.g., Reset Routines for Control) that coaches could introduce for athletes in training and matches, as well as creating 5Cs posters for the Centre walls that staff could point to and use in discussion breaks to facilitate peer-to-peer learning (e.g., ‘How can you regain Concentration when distracted by the score?’).

Subsequently, the coaching staff used weekly IDT meetings to discuss experiences of programme delivery with the SPP, plan the integration of the ‘Cs’ into training and identify opportunities to reinforce psychosocial elements in players’ Individual Development Plans (IDPs). Scaffolding these processes and throughout the 30 weeks, the SP utilised a coaching staff WhatsApp group and provided informal support through face-to-face ad hoc conversations and phone calls.

### 2.8. Individualised Athlete Support

Each athlete completed the 5Cs psychosocial assessment tool (see Figure 1) three times during one-to-one SP consultations across the intervention period. A coach concurrently completed this process for each athlete, which, alongside SPP observations and ongoing feedback from coaches, parents and athletes, constituted individual-level needs analyses. This process subsequently informed the selection of personalised strategies for each athlete (e.g., goal-setting). Individual strategies were selected and modified using a cyclical Teach–Test–Tweak–Repeat approach (Collins & MacNamara, 2017) during one-to-one sessions and in tennis training. Typically, two strategies were selected: one strengths-based, targeting their ‘Super-C’, and another targeting their ‘C for Development’.

**Figure 3:**
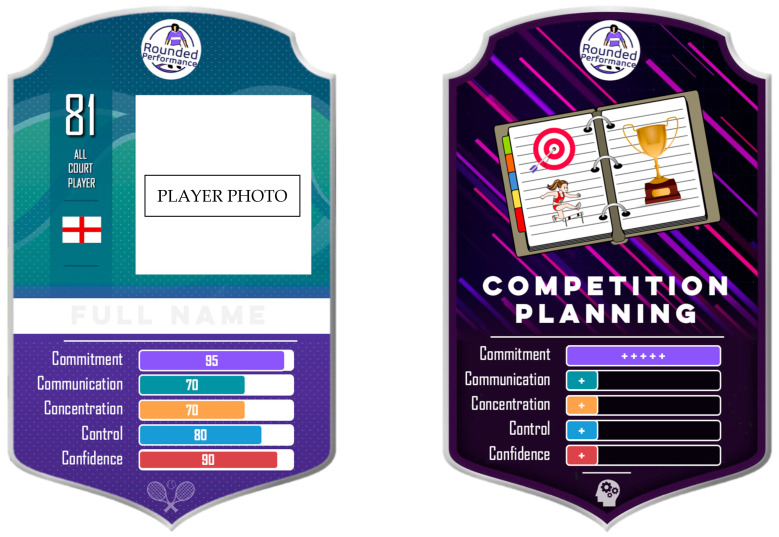
An example Profile Card and Strategy Card.

Each athlete was offered at least seven 30 min one-to-one sessions during the program. When athletes consented, their goals (profiling behaviours) and strategies were shared with coaches and parents to encourage alignment and reinforcement (Steptoe et al., 2019). Athlete strategies were presented to PACs as computer game-styled cards which differentiated player ‘Profile Avatars’, displaying average self-scores akin to Top Trumps^®^ for each ‘C’, and ‘Strategy Cards’, illustrating traditional and novel strategies that could improve behaviour profiles when regularly practised (see Figure 3; Competition Planning for Commitment). Strategy Cards often contained playful analogies and metaphors relevant to individual athletes (Foster et al., 2016; Visek et al., 2015), with athletes able to access the SPP for pre-match phone calls to plan and review their psychosocial goals and strategies for competition. Importantly, coaches also role-modelled engagement with this process by displaying their own Profile Avatar Cards as posters in the gym.

### 2.9. The Parent Development Programme

The parent development programme aimed to provide congruent knowledge and strategies to assist parents in supporting their child’s development and coping with well-documented tennis parenting stressors (see Harwood & Knight, 2009). Two ‘in-person’ workshops were delivered first, focusing on introducing the above programme content and the Strategy Cards system that was linked to the needs analysis process. Secondly, a follow-up workshop targeted the ‘5Cs Parent Journey’, exploring parental stressors, children’s psychosocial development and specific parental strategies to grow the 5Cs (e.g., role-modelling). Following these workshops, the SPP developed and shared a ‘5Cs Parent Job Advert’ poster positioned across a balcony overlooking training courts and operated informal open-door parent drop-in sessions. Parents were also offered formal and informal one-to-one support in-person and by phone.

### 2.10. Procedure

The study was conducted in accordance with the Declaration of Helsinki, and the protocol was approved by the Ethics Committee of Nottingham Trent University (ID: 1853241) on 28th February, 2024. Following ethical approval, both senior coaches at the RPDC were invited to participate and serve as ‘gatekeepers’ by granting permission for the research team to invite parents and their child-athletes. Written informed consent was obtained from adult participants in addition to parental consent forms and child assent forms. Due to the inherent risks associated with qualitative research whereby participants may recognise, and be recognisable by, themselves, each other and third parties, a process of ongoing consent was implemented. This allowed the research team to internally screen extracts case-by-case to identify controversial or revealing information to omit through subsequent collaboration with participants. Through this internal process, however, no data considered for inclusion was deemed compromising, and, therefore, no data was omitted.

### 2.11. Data Collection

The second author, in their embedded role as the SPP, maintained practice-based notes and reflections of their applied work with athletes, coaches and parents, including actions taken with specific 5C strategies to meet individual athlete, coach and parent needs throughout the intervention. These records and actions informed the development of case-based synopses offering insights into the psychosocial development journey taken by athletes and their support teams. At the end of the 30-week intervention period, research data was collected through semi-structured interviews with each athlete, parent and coach separately (m = 77 min for coaches; m = 65 min for parents; m = 53 min for athletes). These were conducted in person by the second author within the RPDC over a three-week period. Based on the research question, the interview schedule focused on understanding the perspectives and experiences of participants in four key progressive areas, with minor modifications made to the wording to align with each stakeholder. Measures were taken to minimise the potential risks of the dual practitioner–researcher role in terms of leading participants to provide positively desirable responses. Specifically, care was taken before each interview to explain that the participant’s involvement would not impact their psychological support or experiences in the program, and that any negatively oriented perceptions or feedback would not be raised post-interview by the practitioner unless the participant wanted to discuss it further. Congruent with this, the practitioner emphasised the benefits of participant candour to the study and prompted participants to reflect upon both positive (i.e., effective) and negative or less effective features as an evaluation of the different elements of the program. Examples of negative case prompts included ‘If you had to change something about the program, what would it be?’, ‘What does the 5Cs model overlook?’, ‘Which of the 5Cs is least important or unimportant to tennis?’, and ‘What parts of the program were not impactful to you?’.

The interview guide itself first asked participants about the psychosocial demands of youth tennis and how positively or fully these were addressed by the program. This led to a series of questions about the value and compatibility of the 5Cs framework with the needs and demands of youth tennis. A third section of the interview explored the roles of parents, athletes and coaches (i.e., the PAC triad) in supporting psychosocial development and behaviour change or enhancement. This transitioned to a final section focused on the implementation and impact of the program. Participants were asked to reflect upon their journey and appraise both the more and less effective programme elements as noted above.

### 2.12. Data Analysis

The analysis procedure employed in this study was reflexive thematic analysis (RTA; Braun & Clarke, 2019). Reflexive TA enabled a thoughtful account of the researcher’s engagement with the data and analytical process, which is both congruent with the assumptions of pragmatism (i.e., the responsibility to interpret participants’ perspectives to produce knowledge most applicable to the research question) and the contextually bound characteristics of case study designs. Closely adhering to the step-based procedure outlined by Braun and Clarke (2019), the second author familiarised himself with the data by transcribing, rereading and relistening to the raw recordings of each participant case to identify rich extracts aligned to the research question. This relates to participants’ perceptions of the relevance attached to the 5Cs framework in terms of psychosocial development in tennis and how their experiences of the intervention offered insight into factors underpinning (in)effective programme features. Semantically similar extracts were grouped to form initial codes within cases, and analysis of patterns between cases enabled the exploration of shared experiences leading to the generation of sub-themes (e.g., personalised to individual needs; provides evidence of growth). These sub-themes then informed the generation of higher-order themes, which aimed to represent the complex interaction between the data, the researcher and their philosophical position, and the research question (Braun & Clarke, 2019). These overarching themes were reviewed against how sufficiently they captured the meaning of coded data and their coherence with the research question. Subsequently, these themes were refined, defined and named as part of a reflexive process with the first author and an additional research colleague who had experience in qualitative data analysis. Through a process of challenging the second author’s interpretations, these researchers acted as ‘critical friends,’ distanced by their lack of involvement in data collection and programme implementation (Sparkes & Smith, 2013). Throughout the study process, additional measures were taken by the second author to enhance research quality (Shenton, 2004). This included maintaining a journal to enhance reflexivity and minimise potential biases. This was particularly important in managing the embedded dual practitioner–researcher role by exploring how prior beliefs about the impact of different programme features and the goodness of fit of the 5Cs framework to tennis could influence data collection and analysis. It allowed for deliberate bracketing of these beliefs through the interview process and informed the search for negative case data throughout analysis. Applied practice notes were utilised throughout the analysis process to check for consistency of PAC accounts with real-world observations. Overall, substantial care was taken to clarify and differentiate the researcher and practitioner roles with participants while maximising the benefits of prolonged engagement with cases through immersion in the TDE.

## 3. Results

Athletes, parents and coaches contributed to the generation of three higher-order themes, each of which represented their perceptions of salient impact factors in the (in)effective implementation of the 5Cs program. These themes reflected the degree to which the programme was (a) *specific to tennis and individualised to the player*, (b) *developmentally collaborative and integrated across the system*, and (c) *evidences growth through accessible and innovative strategies*. The richness of each theme is conveyed below through case participant voices.

### 3.1. Specific to Tennis and Individualised to the Player

This theme reflects the value attached to aligning the 5Cs to the subjective, individual demands and pressures on athletes in relation to tennis as a sport. Participants reinforced the importance of tailoring the 5Cs programme to the specificities of tennis and the different contexts and situations within the sport (e.g., training, competition, match scorelines, poor line calling). They emphasised how the programme had the greatest impact when it targeted those tennis-specific psychosocial behaviours and contexts that athletes found most personally challenging. Coach Eric noted how conceiving the 5Cs in a tennis-specific form allowed him to interweave each ‘C’ with technical and tactical performance:

“Showing Confidence could be a technical thing—having racket speed on the second serve, or it could be their superior body language, or it could be a tactical thing when under pressure—I’m still going to look to execute my wide serve and early pattern.”

While coach Liam appreciated the process of developing the profiling and assessment tool, “The profiling tool is basically what tennis is… I think it takes a little while to identify the behaviours, and it’s always gonna be differently worded, but I think once we got there with this list, when you go through it, it’s pretty much what happens.”

When commenting on the 5Cs framework for tennis-relevant psychosocial characteristics, most participants believed the model ‘*covers*’ the breadth of qualities pertinent ‘*through younger age groups really well*’ and that athletes needed *‘all of the five’* to compete. In addition, participants acknowledged that every player is developmentally different and therefore personalised strategies that were *‘adapted to a specific player’* and one-to-one approaches were viewed as more impactful than group-level strategies and workshops. As Alan commented:

“The 5Cs, you can adapt to a specific player and know what they need to do. It’s a lot more helpful than most things that are built for what is assumed and pre-built… the 5Cs Profiling and the Strategy Cards, it really captures [the needs of] the specific player.”

Tom’s mother, Clara, supported this by stating:

“It puts everything you have learned into a structure; what you need to have in a child, and in yourself, to be successful in that sport… The 5Cs were easy to remember. They were exactly what I saw before every single serve, every single return on the court that he had to go through… it put the structure into what I was seeing, and then we could work on these.”

Participants noted how the psychosocial programme was effective in helping PACs to develop strategies to cope with the stressors of the tennis system and the different situational demands. They proposed how athletes needed to be supported in demonstrating and executing psychosocial behaviours specific to various tennis scenarios, when playing specific opponents, at different scorelines and depending on tournament grades. Athletes and parents, in particular, shared how the importance of each ‘C’ related to the specific context or scenario. Most participants believed it was harder to demonstrate Confidence, Commitment and Control in competition, whereas Concentration was more heavily challenged by distractions at training, where the intensity could be lower. As Alan shared:

“In training, it will be easy to keep your confidence, commitment and control because, I mean, you’re not very mentally tough if you’re beating yourself up and getting really pissed in training, but it might be a bit harder to keep concentration because you’ve not got the drive.”

All participants shared beliefs that Communication was relevant and impactful in terms of appropriately interacting with opponents and doubles partners, but was less important than other ‘Cs’ in terms of performance execution on-court. However, it maintained a more elevated significance off-court to plan and reflect with coaches and align PAC’s approaches to support the wider development of athletes. Tom reinforced this, stating:

“I think communication is still an aspect of tennis, but it’s just not as important as control, concentration and confidence. Maybe planning what you’re going to do before a tournament with a psychologist or a coach. … after the match as well, or when you’re home, I think communication is probably more important than it is on court because you’re not allowed to really communicate in competition.”

Several parents reinforced how it was important to train each ‘C’ under situation-specific and competition-like pressures because they noted how their children struggled most with Confidence, Concentration and Control when their child faced certain opponents and scorelines. As Rhea’s father, Devansh, summarised:

“They might be concentrating very hard, but their confidence level may be shattered seeing the opponent if they know that the opponent is probably a stronger player or if they are playing a very weak player [and] they know that you are going to win, then their concentration level can go down, or if they are playing in a Grade One, they might get nervous in a bigger stage, and then confidence can drop as well.”

Where psychosocial behaviours and strategies such as goal-setting, routines, positive body language and reflection are not extensively trained in competition-like situations in practice, the programme risked being less effective. Reflecting on the need for further conditioning, Chloe acknowledged: “We’re giving him the tools to be able to focus on the right thing at the right time, but he doesn’t seem to be able to utilise them at that endpoint in a competition environment… he does appear to be trying to make use of those tools. It might just be that it’s going to take you more time to get there”.

### 3.2. Developmentally Collaborative and Integrated Across the System

This theme captured the importance of factors that helped to embed psychology within the TDE to provide the structure, clarity and strength of support needed for each athlete to develop. This also included the need to ensure that parents maintained appropriate developmental expectations around the 5Cs and their child’s progress.

Firstly, coaches shared how collaboratively defining the 5Cs and embedding the programme into the RPDC environment through amplifying voices across PACs was key to garnering programme buy-in. Both coaches noted how conceptualising and tailoring each ‘C’ to the tennis context, and aligning behavioural expectations for the athletes’ support network, was key. Eric stated:

“Involving staff in the selection of valued behaviours is a bit like the players having ownership with it, so it feels a bit more like it’s our own identity, our own thing… The buy-in from the coaching staff is then high because we’re delivering what we believe in.”

Liam affirmed:

“It was a really smart profiling tool to get parents and athletes to buy into something that’s quite difficult to feel… Where the crossover becomes really good is the link of parents’ understanding of behaviours and the goals. The players had an idea of “these are the things we need to be good at to be a certain tennis player”. We were now using similar language, and they are way more skilled, a bit like us… They were definitely better at going; “My concentration wasn’t quite as good, and I need to do X, Y and Z.”

Shared understanding of the 5Cs and individualised athlete strategies empowered parents to take an active support role by providing reminders, facilitating reflection and reinforcing efforts to improve. This role was typically enacted around competitions when coaches were absent. Shreya’s mother, Leila, identified:

“Having that competition planning sheet is important for us… it’s probably given us a bit of like, “Alright, okay, let’s talk about this”, “Let’s set your goals”… and just trying to reinforce stuff that she’s maybe talking to you and the coaches about.”

Parents and coaches emphasised the impact of upskilling parents to cope with psychosocial challenges they experienced while ‘co-journeying’ through tennis. Parents believed they *‘needed as much support as the kids’* and that workshops, drop-in sessions and remote competition support from the SP not only aligned core messages around development but were reassuring, encouraging and helped to establish a sense of parental community in the RPDC. Clara disclosed:

“The workshops for parents, informal and formal chats and the remote support helped…. Other parents shared what they felt, and I thought, “Yeah, I feel like that!”. But you don’t talk about that with others because you always think you’re giving away your top secrets, your weaknesses… We had good conversations after that evening. I was working with the other parents and felt more connected with them.… I’m feeling much better because I know if I’m not feeling good, I can call you [the SP]… I don’t want to leave that with a coach… I don’t want to discuss it with my husband because then we argue… I can’t talk to Tom about how I feel because I don’t want to worry him… Through you as the SPP, I’m getting constructive help to deal with situations… I tell Tom “When you want to talk to me about something, you talk about that. If you don’t want to, it’s okay, you’ve got [SPP]”.

Coaches equally noted that they had *‘no concept in my head of how to deliver or coach mental skills’*, but now valued the *‘need to be skilled in that area’* to proactively coach the 5Cs in sessions and at tournaments. Coaches cited how team meetings, bite-sized resources and informal chats improved their confidence in integrating psychosocial principles into their practice, ultimately impacting athletes. Eric shared:

“Because of how simple the 5Cs is and because of our little team meetings and conversations…I think that’s why players are responding to it better; because it’s coming at them from all angles a bit more consistently… those booklets that you put together for us are easily digestible for coaches to jump on court and deliver”.

[Eric continues] “In matches, you’ve got one sentence to try and impact what they’re doing, whether it’s the change of set or before the next serve. Whereas at training, you’re building that body of work about what that word means… At tournaments, it’s really easy to go ‘come on concentrate’, or ‘fully commit here’. To each player, that might mean something different because of what they’ve been working on in their individual goals… That’s been achieved through the way it’s been delivered; in the way the model has been presented to us and integrated”.

The integration of the SPP on court with coaches emerged as a sub-theme with a strong consensus across cases. Viewed as normalising psychology, participants believed that the SPP served as a collaborative ‘glue’ that influenced positive interactions in the PAC triad. Tom shared how “whenever you’re there to help with psychological things, I think it’s also quite useful… because then you can kind of guide us to the right things to do”, with Liam reinforcing that “Support at training—that’s a big one for me… If we hadn’t had that, there would be a much smaller buy-in… Coming from a playing background, anything that wasn’t on court, especially that age, I didn’t find relevant”.

Reflecting the value of the SPP on court and how 5Cs strategies in training helped match preparation, Eric stated:

“It’s not mental skills over here, technical over there; everything comes together and that’s where it becomes more powerful. That’s why it’s been really effective, we’ve seen players improve a lot in these 5C areas, match performance and results because the gap between mental skills and tennis has merged. So, it’s very easy for everything to be reinforced in the gym, with you, on court, and at the match. The clarity and the crossover is what’s actually the best thing about it.”

Insights were provided by participants into factors that rendered the programme less effective, or which might have constrained effectiveness. Coaches believed that where parents held developmentally unrealistic expectations of their child’s development and demanded more short-term results, they bought into and engaged with the programme less. They believed the programme needed to sell a *‘long-term look’* to parents even more strongly, to stress the importance of ‘*keeping the journey in perspective’*, and anticipating *‘up-and-down’* development as players ‘*physically and mentally mature’*. Some parents, however, noted how the programme had helped them become more developmentally sensitive. Clara reflected:

“It was like an apprenticeship from being absolutely poor… it just trained me how to be a good parent for him… It’s a long journey with these mental programmes. I find some parents I’ve spoken to here expected quicker wins. That doesn’t come because the child’s brain has to be mature enough to take these things in… They look really grown up but don’t get fooled… as a parent, when somebody told me why they can’t physically do that—they can’t physically control that, that’s when the penny dropped, and I could accept that behaviour more”.

Devansh further reinforced: “What you learn as a parent is to keep your expectations low, keep supporting your child, and give them a bit more Confidence and [help them to] Control their emotions as well, which is very hard to do… So you have to control your emotions as well”. A final, relevant observation and recommendation was parents having five sequential workshops on each ‘C’ co-ordinated with the player workshops, or joint workshops together, as Eric suggested:

“If the players are having five [workshops] on each of the five C’s, the parents are either involved in that or have their own separate ones [for] their understanding of the behaviours and what we’re expecting with players… If they’re a bit more on the same page, hopefully that learning happens a bit faster.”

### 3.3. Evidences Growth Through Accessible and Innovative Strategies

This final theme represents the perception that the effectiveness of the 5Cs programme was dependent on the ways it could evidence improvement and the user-friendly strategies and tools that captured the imagination of the athlete. It also integrates reflective perceptions of the use of the profiling tool and applied case notes as part of the process of evidencing improvement. Firstly, participants praised the accessibility, structure and age-appropriate language, believing that psychology was made more appealing by visual cues that reminded them of the 5Cs and strategies to develop them. For example, the 5Cs parent job advert posters, Strategy Card bag-tags and stickers on tennis rackets were believed to improve engagement with the programme content. Particularly engaging to athletes were the ‘gamified’ Strategy Cards, as Tom shared: “It kind of gamifies it a bit so it looks like a FIFA card. And I think it’s a fun way of choosing what things you work on and which of the 5Cs is your strongest point and your weakest one.” Liam supported this by affirming:

“I think the cards have been a really good way to introduce psychosocial strategies with younger age groups… in an easy, accessible, understandable way so that they have fun doing it and integrating in…They were quite proud of having it first and foremost; it was something they wanted to show off… Little kids need reminding way more often… having a card really highlighted the mental strategy hanging off the back of your bag… If someone came onto the court for the lesson, that just reminded me when I saw it hanging outside: “I’m working on World Class Acting today!”

It was important to parents and coaches, however, that the 5C strategies led to evidence-based improvements, with such perceptions frequently derived from either observing improved athlete responses or use of the relevant strategies. Discussing changes she had noticed in her son’s behaviour, Brian’s mother Jade shared:

“I think this probably comes into Control—the reset. That’s what I think he’s developed the most. I’ve seen him do a bit of the breathing and the reset routine, making sure he uses his towel and stuff like that… Mr Bobblehead, that was a good Strategy Card, and he doesn’t show that [negative body language] quite as much.”

Eric offered their views on a number of athletes, stating:

“It’s noticeable in their reset routines, goal-setting, reviewing their games, and the behaviours they’ve been working on; they’re visibly doing these things. As a result, they’re more resilient competitors… they’re coming through difficult things and winning at a level they weren’t doing before… Shreya having her best results over this last period.… Rhea, who’s having more ups and downs, is still probably competing at the best level she has—she’s had National Camp selection … The impact on [a non-participant athlete] has been huge; she was struggling to get over the line in some of the important moments and matches and she won an ITF in four months… Them being better is born out of doing those behaviours more… using a towel, taking more time doing those things… they’re more equipped with loads of tools and they’ve got a much larger understanding of those tools and why they’re doing them.”

Participants gave particular weight to strategies designed to reduce the incidence of unpleasant internal cognitive and affective experiences, and for many Control was viewed as the ‘C’ for greatest development. As individual Strategy Cards targeting anger and nerves on court, Brian shared how “Mr. Bobblehead helped, and the Reset Routine as well… it helps my anger.”

Participant perceptions of programme features, whether as effective or ineffective in terms of improving awareness, strategy use or performance, were typically supported by the practitioner case notes. For instance, in Brian’s case, the PAC recalled improvements in his ability to discuss his performance of the 5Cs (e.g., the effect of Control on his response to anger) and execute strategies (i.e., reminding himself to manage ‘Mr Bobblehead’ as part of a ‘Reset Routine’) but with no notable effects on bouncing back in subsequent points. These insights were supported by practitioner notes and observations, which highlighted improvements in his initial responses to setbacks, yet a continuation of rash decision-making in subsequent points.

Considerable variability was, however, observed in the trajectory of athletes’ profiling scores across the programme, with patterns inconsistently aligned with the accounts from PACs of improved athlete ‘C’ behaviours. Practitioner case notes indicated that the variability in athlete self-scores was partly attributable to limited athlete self-awareness early in the programme; this was identified by the practitioner at the time of profiling to have risked inflated self-reporting compared with his own observations. In Rhea’s case, reductions in athlete self-scores were interpreted in case notes as reflecting heightened self-awareness and effective psychoeducation rather than behavioural regression, a pattern that aligns with participants’ accounts of her growth in this study. Practice notes also captured reflections from Shreya that her progression through increasingly challenging grades of tournaments had changed her reference points for her scores, which explained her self-score variability. Case notes also documented inconsistencies in the coach-completed profiling of players between timepoints due to their availability and workloads. This influenced the reliability of coach-reported profiling scores. Overall, practitioner case notes provided greater context that supported participant accounts of growth while illustrating why profiling data often suggested variable trajectories and served more effectively as a stimulus for conversations and perspective sharing.

As a final key insight, all participants emphasised that the Cs were ‘linked’ to each other, and a number of athletes therefore assessed how the programme had worked across multiple Cs, including their perceived strengths and weaknesses. Rhea closes our results by noting:

“All of the Cs have been good so far in my tournaments… It’s not perfect, obviously, but I’m doing everything way better… I remember that I used the ‘Sponge Human’ and ‘Reset Routine’ cards in my match… they made me better… by making me feel more controlled and relaxed… Sponge Human was mainly a concentration strategy, concentration plus communication. Yeah, about communicating with yourself… Concentration, well, that’s one of my worst Cs, but I try to look at my strengths as much as I can. My Reset Routine helped me to be calm, focused, and clear to bounce back with energy… I’ve been better at that… and at what happens next… I play better.”

## 4. Discussion

This study explored athlete, parent and coach experiences of a 5Cs psychosocial development programme in elite youth tennis with a view to identifying critical impact factors in relation to programme relevance and effectiveness. Bridging a gap in the applied youth tennis literature and extending the application of the 5Cs framework beyond youth football, the study facilitated a rich and revealing understanding of how to most effectively implement psychological content to influence the development of salient psychosocial characteristics in young players (Dohme et al., 2019; Harwood, 2008; Lauer et al., 2020).

A primary outcome of the study was the promising validation of the 5Cs framework as a structure of concepts that resonated with athletes, coaches and parents through their alignment with the psychosocial demands of the sport. By tailoring the framework to the sport’s unique demands and engaging in a collaborative process to identify valued behaviours and qualities, the SPP practitioner was able to create and promote a shared language that made psychosocial concepts accessible and actionable. The profiling tool informed by these behaviours helped to facilitate further buy-in and enabled the SPP and PACs to work cohesively together on maintaining psychosocial strengths and targeting areas for development (Steptoe et al., 2019).

Beyond coaches, interview data from athletes and parents illustrated their understanding of the 5Cs and how confidently they could locate the importance of the ‘C’ and its behavioural properties in the context of the stressors and situational demands of tennis. The ego-involving, result-oriented nature of the sport (Nicholls, 1989; Harwood & Knight, 2009) meant that Control, Concentration, Confidence and Commitment-related behaviours and characteristics were believed to be especially tested. While on court, match-based Communication with opponents and doubles partners was valued less than other ‘Cs’ (i.e., ‘Help’, ‘Encourage’, ‘Praise’; see Harwood & Anderson, 2015), participants attributed high importance to information-receiving skills during on-court practice and those interpersonal skills required to plan and reflect off-court with coaches and parents (i.e., ‘Listening’ and ‘Acknowledging’; Harwood & Anderson, 2015).

Through programme implementation, several factors emerged from the data that offer direct practical messaging to sport psychologists as to ways of optimising the effectiveness of a psychosocial intervention in a TDE. Firstly, individualisation to the personal needs and goals of the young athlete is a cornerstone of effective psychosocial support. While group-level workshop delivery initiated and facilitated a shared knowledge base and common language on court, athletes and parents respected the individual Profile and Strategy Cards, the one-to-one sessions, ad hoc conversations and wider support availability of the practitioner for their child. Collins and MacNamara’s (2017) cyclical Teach–Test–Tweak–Repeat approach proved important to athletes as an autonomy-supportive method, which encouraged their responsibility to modify strategies and communicate with the practitioner. Such individual attention was critical for perceived impact, given that athletes and parents tended to deviate towards areas for improvement over existing psychosocial strengths, and such needs were specific and contextual to different players. This close consideration was also important in contextualising the profiling scores, where improvements may have existed between timepoints but were less objectively apparent due to over-rating at baseline or inconsistencies in coach completion.

Secondly, by conceptualising each ‘C’ as characteristic behaviours which could be improved by parent, coach and athlete strategies, PACs were empowered with distinctive but complementary support roles in working towards the athlete’s ‘C’-related goal (Dohme et al., 2019; Tagliavini et al., 2024; Wolfenden & Holt, 2005). The salience and effects of collaboration and communication within PAC triads reinforce Tagliavini et al.’s (2024) findings that youth tennis players benefit from interpersonal relationships where parents and coaches deliver consistent messages and reinforce strategies in a child-centred, coach-led and parent-supported system (Horne et al., 2020; O’Donnell et al., 2022; Thrower et al., 2023). The 5Cs framework appeared to facilitate high-quality relationship functioning through the use of innovative strategies and gamified tools that bonded with the imagination of all stakeholders, and were particularly in tune with the developmental stage of the athletes (e.g., Profile Cards, Strategy Card Bag Tags; Thrower et al., 2024).

Underpinning the collaborative support role of tennis parents was simple recognition by the SPP and coaching team that they were a *resource to be optimised* as opposed to a *problem to be distanced* by the TDE (Horne et al., 2020; Tagliavini et al., 2024). Disrupting the enduring and oversold narrative of ‘pushy and pressurising sport parents’, an effective psychosocial programme embraced the need to support and upskill parents as developing experts on their child who provide critical support at tennis competitions in the absence of coaches. Aligning with Harwood and Knight’s (2015) postulates of sport parenting expertise, parents appreciated the educational workshops and engagement with the SPP that enabled them to better manage the isolating demands of tennis and model appropriate support to their child through shared athlete–parent discussions and developmentally realistic expectations.

An important caveat here emerged through participants reinforcing that programme effectiveness depended on the SPP raising parents’ awareness of stage-appropriate behavioural expectations and more assertively challenging parents to look beyond quick-fix ‘psychological first aid’ for short-term issues. Development was less evident for players whose parents limited their engagement in the programme, and who prioritised a focus on results over process goals. However, by using a small number of developmental goals targeting player-specific behaviours and strategies (e.g., use of Reset Routine for Control) in training and competition, the SPP created opportunities for the athlete to evidence observable improvements related to a ‘C’. This energised further commitment from parents who could see the visible signs of engagement and improvement in their child.

A third critical factor arising from participants’ accounts was the quality of the coach–SPP relationship, not only in terms of engaging coaching staff ‘buy-in’ through the relevance and accessibility of the 5Cs framework, but also in terms of on-court collaboration between SPPs and coaches through session design and strategic integration. Akin to findings within academy football (e.g., Harwood, 2008; Harwood et al., 2015), the pedagogical utility of the framework was pivotal to embedding psychology through coaching to impact positive responses and behaviour in training. The multimodal approach to coach education, including PROGRESS resource packs, IDT review meetings and ad hoc conversations with the SPP, maintained the momentum of implementing the 5Cs blocked curriculum. Interestingly, the on-court presence of the SPP at training proved important to building rapport and such care and investment positively engaged the athletes (Foster et al., 2016; Johnson & Gilbert, 2004). Our findings highlighted that SPP involvement alongside coaches can normalise psychology and support knowledge transfer from psychoeducational resources, workshops and one-to-one sessions onto the court by reinforcing messages, strategies and positive coach and athlete behaviours (Dohme et al., 2020; Lauer et al., 2020).

### Future Research Recommendations and Practical Implications

This study is not without limitations and constraints, yet examining these presents constructive research and applied opportunities. Firstly, the dual scientist–practitioner role played by the second author risked participants presenting in a way they felt would appease the researcher due to their prior SPP relationship. However, as articulated earlier, great care was taken to emphasise and differentiate the researcher and practitioner roles to participants, and highlight how critique would benefit future support. Participants provided rich and candid experiences, including recommendations to sell the 5Cs framework better as a strengths-based developmental tool to parents obsessed with short-term outcomes. In addition, coaches and parents proposed having five sequential workshops on each ‘C’ for parents, or for parents and children together, aligned to the blocked curriculum with coaches. We believe that the scientist–practitioner component served the research question in this particular study effectively due to the relationships developed. However, future intervention designs with SPPs employed in Talent Development Environments may benefit from an external, independent researcher responsible for participant data collection and analysis.

Secondly, while specific subjective details emerged on the importance of certain ‘C’-related strategies (e.g., Reset Routines) and the salience of creating competition demands and conditions in practice, the study was limited in its objective observations of parents, coaches and athletes at training and competition. Using the platform of our findings, future observational research could investigate which PROGRESS strategies coaches most frequently deploy to develop specific ‘Cs’, and, indeed, which coaching strategies athletes felt served as the greatest source of confidence and development in that particular psychosocial behaviour. One particular research opportunity here, given the adolescent stage of these athletes, might be to investigate the impact of peer-to-peer learning and how well creating a climate that promotes and reinforces peer support on court may serve to accelerate psychosocial development.

Thirdly, the education and support provided to parents empowered them to grow their sport parenting expertise (Harwood & Knight, 2015) and play active roles in helping their child to set psychosocial goals for competition (e.g., take time to breathe between points using your towel to keep your Control battery charged). However, future research could target how the pre-match process of athlete–parent 5Cs goal setting, observation and post-match discussion influences maintenance and improvements in psychosocial strengths over time. This is particularly relevant in tennis because parents are often the consistent attendees at competitions, as coaches tend not to observe matches until an athlete reaches a higher level.

Finally, a fundamental gap in the research here is the extent to which psychosocial qualities and behaviours developed through sport transfer to other non-sport, life domains (e.g., behaviours in school, family life, community, social activities; Mossman et al., 2021). Prior research into 5C programmes has provided subjective evidence of transfer effects to domains such as the classroom (e.g., greater confidence in public speaking; Harwood, 2008; Kramers et al., 2023). However, while such programmes took an intentional and explicit approach to psychosocial development in the sport, they lacked a deliberate and planned ‘dual’ goal emphasis, which is necessary for ‘purist’ research into life skills transfer (Bean et al., 2018; Mossman et al., 2021). In such research, deliberate and intentional efforts need to be made for promoting psychosocial behaviours within and through the sport, with active and integrated roles played coaches, parents, athletes and teachers (for example) on promoting, linking and reinforcing the demonstration of congruent ‘C’-related behaviour in the non-sport setting (e.g., Commitment—showing perseverance in a new subject at school). Future researchers and practitioners focused on holistic psychosocial development through a TDE may consider a programme that progresses participants through the ‘knowing, growing and showing’ phases of the 5Cs. In this initiative, athletes are introduced to the 5Cs and valued behaviours are identified (knowing phase); individual needs analysis then leads to goals and actions to maintain and improve strengths and target areas (growing phase); and, finally, athletes and their support team identify psychosocial strengths to transfer and ‘show’ in other non-sport contexts (showing phase). Such a design may not only contribute to the limited body of life skills transfer literature in sport (Mossman et al., 2021), but also help to improve the integrity and responsibility of organised high-performance sport cultures vis-à-vis their duty of care to young people and prevention of athlete mental health issues (DCMS, 2017). In concert with Hauser et al.’s (2024) insights into functional features of optimal TDEs, the 5Cs may serve as the facilitatory framework for holistic and integrated efforts to promote child-centred development within the organisational culture.

At an organisational level, the findings of our study suggest there is a strong appetite and need for embedded sport psychology provision for youth tennis environments. Given that many RPDCs remain without the funding needed to embed SPP support, we encourage the LTA national governing body to reflect on the current lack of funded psychological provision across the British youth tennis system, its duty of care to young players, and consider whether it adequately supports PACs in coping with the psychosocial demands of the sport. By integrating developmentally relevant content to athletes, mediums such as on-court training, scenario-based examples, analogies, metaphors, visual aids and tangible, gamified accessories represent contexts and strategies that seem to best attract and influence athletes towards desired psychosocial development (Foster et al., 2016; Visek et al., 2015). We hope that this study offers a blueprint of processes and ideas for both organisations and sport psychology practitioners to effectively work with young athletes and their support teams in tennis and beyond (Thrower et al., 2024).

In conclusion, implementing the 5Cs framework through a multimodal psychosocial development programme within competitive youth tennis was perceived as impactful in developing tennis-relevant psychosocial characteristics and behaviours. The utility of the framework was underpinned by the degree to which it offered tennis-specific, individualised support; engaged coaches, parents and players in collaboration; and was able to evidence improvements through accessible and innovative strategies. We encourage researchers and practitioners to extend these findings, and organisations to employ SPPs in youth sport environments to ensure that a child’s psychosocial development is appropriately cared for.

## Figures and Tables

**Table 1:** Participant cases and demographics.

Case(s)	Pseudonym	Role	Age	Gender	Ethnicity
1	Rhea	Player	13	Female	Asian Indian
	Devansh	Father	46	Male	Asian Indian
2	Alan	Player	13	Male	White British
	Chloe	Mother	52	Female	White British
3	Tom	Player	14	Male	White British
	Clara	Mother	45	Female	German
4	Brian	Player	11	Male	White British
	Jade	Mother	42	Female	White British
5	Shreya	Player	12	Female	Asian British
	Leila	Mother	46	Female	Asian British
All	Eric	Coach	39	Male	White British
All	Liam	Coach	39	Male	White British

## Data Availability

The original contributions presented in this study are included in the article. Further inquiries can be directed to the corresponding author.
